# Remote Monitoring for Rheumatoid Arthritis Flare During Drug Tapering: Protocol for a Prospective Observational Cohort Study

**DOI:** 10.2196/99594

**Published:** 2026-09-21

**Authors:** Javad Sarvestan, Marzieh Shahmandi, Najla Elndari, James MS Wason, Silvia Del Din, Kenneth F Baker

**Affiliations:** 1 Translational and Clinical Research Institute, Faculty of Medical Sciences, Newcastle University, Newcastle Upon Tyne, England, NE4 5PL, United Kingdom; 2 National Institute for Health and Care Research (NIHR), Newcastle Biomedical Research Centre (BRC), Newcastle University and The Newcastle upon Tyne Hospitals NHS Foundation Trust, Newcastle University, Newcastle upon Tyne, England, United Kingdom; 3 Biostatistics Research Group, Population Health Sciences Institute, Faculty of Medical Sciences, Newcastle University, Newcastle Upon Tyne, England, United Kingdom; 4 Rheumatology Department, The Newcastle upon Tyne Hospitals NHS Foundation Trust, Newcastle University, Newcastle Upon Tyne, England, United Kingdom; 5 Translational and Clinical Research Institute, Faculty of Medical Sciences, Newcastle University, Room M4.089, 4th Floor William Leech Building, Medical School, Newcastle Upon Tyne, England, NE2 4HH, United Kingdom, 44 7986733128

**Keywords:** rheumatoid arthritis, disease-modifying antirheumatic drugs, DMARD tapering, arthritis flare, telemonitoring, wearable sensors, accelerometry, Axivity AX6, digital biomarkers, physical activity, mobility monitoring, sleep monitoring, patient-reported outcome measures, Rheumatoid Arthritis Flare Questionnaire, prospective observational cohort study, eHealth, digital health, joint modelling

## Abstract

**Background:**

Rheumatoid arthritis (RA) disease activity during disease-modifying antirheumatic drug (DMARD) tapering is commonly monitored using in-person clinical assessment and the 28-joint Disease Activity Score with C-reactive protein (DAS28-CRP). Although effective, this approach is resource intensive and may be inconvenient for patients. Remote monitoring with patient-reported outcomes and wearable sensors may enable earlier flare detection and support safer, more personalized tapering pathways. Prior pilot work suggests that accelerometry-derived physical activity, mobility, and sleep metrics correlate with RA disease activity and are acceptable to patients.

**Objective:**

This protocol aims to evaluate the feasibility and diagnostic accuracy of remote monitoring for detecting RA flare during DMARD tapering. The study will (1) continuously measure physical activity and quality metrics using wrist-worn accelerometers, (2) collect weekly Rheumatoid Arthritis Flare Questionnaire (RA-FQ) scores, (3) develop and evaluate dynamic risk prediction models using longitudinal accelerometry-derived measurements to estimate the risk of clinically confirmed flare, (4) retrospectively assess prediction accuracy against patient-reported flare, and (5) develop and evaluate a combined dynamic prediction model incorporating longitudinal accelerometry-derived measurements and total RA-FQ scores to assess whether the addition of the RA-FQ improves the prediction of clinically confirmed flare.

**Methods:**

This prospective, single-center observational cohort study is embedded within the Rheumatoid Arthritis DMARD Tapering (ROADMAP) clinic at Freeman Hospital, Newcastle upon Tyne, United Kingdom. Adults with clinician-confirmed RA in remission who are undergoing or are about to begin DMARD tapering will be recruited, with a target sample of 100 participants. Participants will be followed for 12 months, with clinical assessments aligned with routine ROADMAP visits at baseline and approximately months 3, 6, 9, and 12 together with ad hoc visits for suspected flare. Continuous wrist-worn accelerometry and weekly RA-FQ responses will be collected throughout follow-up. Clinical assessments will include tender and swollen joint counts, patient and physician visual analog scales, C-reactive protein, the DAS28-CRP, and Health Assessment Questionnaire Disability Index scores. The primary outcome is time to first clinically confirmed flare, defined as DAS28-CRP≥2.4 and/or at least 1 swollen joint attributable to inflammatory RA activity. Dynamic risk prediction methods, such as landmarking models, will use longitudinal accelerometry-derived physical activity, mobility, gait, and sleep measures to estimate flare risk over prespecified prediction horizons. Model performance will be assessed using time-dependent discrimination, calibration, predictive values, and lead time before clinically confirmed flare.

**Results:**

The first participant was recruited in October 2025. As of July 2026, 16 participants had been recruited. This protocol reports the study design and planned analyses; outcome analyses will be conducted after recruitment and 12-month follow-up are complete.

**Conclusions:**

This study will provide pilot evidence on the feasibility and accuracy of multimodal remote monitoring for RA flare detection during DMARD tapering in routine care. Findings will inform model refinement, external validation, and future larger multicenter studies evaluating clinical utility and service impact.

## Introduction

Rheumatoid arthritis (RA) is a common disease affecting approximately 1 in 100 adults in the United Kingdom [1] and is characterized by joint pain and swelling and general features such as fatigue [2]. Patients with RA are prescribed disease-modifying antirheumatic drugs (DMARDs) in order to prevent joint damage and improve symptoms [3]. With modern treat-to-target DMARD regimens, approximately two-thirds of patients newly diagnosed with RA can now achieve disease remission. Once remission is achieved, international clinical guidelines now endorse the gradual reduction (tapering) of DMARDs to reduce the risk of drug side effects and toxicity, reduce medication burden for patients, and reduce drug prescription costs.

The Rheumatoid Arthritis DMARD Tapering (ROADMAP) service was established in early 2023 at Newcastle Hospitals to support a consistent, standardized approach to DMARD tapering for patients with RA seen in rheumatology outpatient clinics [4]. Patients may enter the service while receiving either a full therapeutic dose or a previously reduced but stable dose; therefore, enrollment in this service is not restricted to patients beginning their first tapering step. Tapering is individualized according to DMARD class, current dose, duration of remission, previous tapering history, and clinical judgment. Dose reductions are generally introduced stepwise at approximately 3-month intervals rather than through an immediate reduction (eg, to 50% of the original dose) [4]. At each visit, the current dose, proportion of the reference therapeutic dose, date and magnitude of the most recent reduction, and planned next tapering step are recorded. If flare occurs, treatment is returned to the previously effective dose, with short-term bridging glucocorticoids used when clinically indicated.

In routine clinical care, RA disease activity is commonly evaluated using a composite measure based on tender and swollen joint counts, an inflammatory biomarker (C-reactive protein [CRP] or erythrocyte sedimentation rate [ESR]), and a patient-rated visual analog scale, which together generate the 28-joint Disease Activity Score (DAS28) [5-7]. Because this assessment typically requires in-person hospital visits, ongoing monitoring can be resource intensive and costly [8,9]. In addition, patients in sustained remission are often reviewed infrequently (typically annually), meaning that scheduling 3-monthly outpatient appointments during DMARD tapering can be inconvenient for patients and may represent an inefficient use of clinician time and hospital resources.

Compared with DAS28-based monitoring, several patient-reported outcome measures (PROMs) have been developed to identify RA flare using self-completed questionnaires that capture multiple dimensions of disease activity, including fatigue, pain, mood, and physical function [10]. One widely evaluated instrument is the Rheumatoid Arthritis Flare Questionnaire (RA-FQ), which has been endorsed by the international OMERACT consortium for this purpose [11]. PROMs can be completed remotely without hospital attendance and can capture subjective aspects of disease impact that are not directly represented within the DAS28 framework. In parallel, advances in accelerometry have enabled wearable devices that remotely measure and quantify physical activity and gait characteristics [12]. In prior work, wrist-worn and lower back–worn accelerometers were used to derive activity and gait metrics in 11 patients with active RA who were newly initiating DMARD therapy [13]. These pilot findings indicated strong correlations between accelerometry-derived outcomes and clinical indicators of disease activity, particularly inflammatory biomarkers (CRP and ESR) and tender joint count. Patient feedback also suggested high acceptability of the devices, with a preference for the wrist-worn placement [14]. To this end, the main objective of this study is to demonstrate the feasibility and accuracy of accelerometry monitoring for the detection of RA flare during DMARD tapering. Primary and secondary subobjectives of the study are presented in Textbox 1.

Textbox 1. **Primary and secondary objectives of the study.**

**Primary subobjectives**

1. Predict clinically confirmed flare risk using dynamic risk prediction methods based on longitudinal accelerometry-derived measurements
2. Assess retrospectively the accuracy of the dynamic risk prediction models for flare prediction vs patient-reported flare

**Secondary subobjectives**

1. Assess the usability and acceptability of continuous accelerometry monitoring using patient feedback questionnaires
2. Assess retrospectively the accuracy of Rheumatoid Arthritis Flare Questionnaire (RA-FQ) flare prediction vs clinically confirmed flare
3. Develop and evaluate a combined dynamic prediction model incorporating longitudinal accelerometry-derived measurements and the RA-FQ total score to predict clinically confirmed flare and compare its performance with that of the accelerometry-only model

## Methods

### Study Design

This prospective, single-center observational cohort study is embedded within the ROADMAP DMARD tapering clinic at Freeman Hospital, Newcastle upon Tyne, United Kingdom (Multimedia Appendix 1). Adults with clinician-confirmed RA in remission who are undergoing or are about to begin DMARD tapering will be followed for 9 months. Continuous wrist-worn accelerometry and weekly RA-FQ responses will be collected throughout follow-up, with clinical assessments aligned with routine ROADMAP visits at baseline and approximately months 3, 6, 9, and 12 together with ad hoc visits for suspected flare.

### Ethical Considerations

Patients will be required to attend face-to-face hospital visits on a maximum of 3 occasions during study follow-up. To avoid additional study visits beyond standard clinical care, we have aligned the study visits with the clinic visit schedule of the ROADMAP clinic, thus avoiding additional trips to the hospital for participants [13,14]. Under the currently approved protocol, participants may be followed for up to 6 months. An amendment has been submitted to the Health and Care Research Wales Research Ethics Committee (REC) to extend the maximum follow-up period by an additional 6 months, from 6 to 12 months. The amendment is currently under review by the same REC. The extended follow-up procedures will not begin until a favorable ethical opinion and all required sponsor and site approvals have been obtained.

The study was approved by the Health and Care Research Wales REC (reference 25/WA/0043). All participants will provide written informed consent before enrollment, and additional consent will be obtained for the extended follow-up where required. The study will be conducted in accordance with the Declaration of Helsinki, and it was prospectively registered with ISRCTN (ISRCTN14472047) before the recruitment of the first participant. The completion of the weekly RA-FQ through the REDCap portal requires access to a computer or mobile device, internet access, and sufficient digital literacy. To reduce exclusion related to digital access, participants unable to use REDCap will be provided with paper RA-FQs.

Tapering of DMARD therapy is associated with a risk of arthritis flare. In our previous research studies and based on outcomes to-date from the ROADMAP clinic, the risk of flare is approximately 50% [15]. Importantly, the patients in this study taper their DMARDs as part of routine clinical care within the ROADMAP clinical service, which lies outside the scope of this project protocol. Patients are informed of this risk, which is discussed in detail during their initial ROADMAP clinical consultation prior to participation in this study and thus does not form part of the ethical considerations of this study protocol.

### Target Recruitment Sample Size

This is explicitly a development project phase, and as such the analysis is intended to produce pilot data to support and inform the design of a future larger definitive trial of remote monitoring in DMARD tapering. To determine the required sample size for the current study, our primary focus lies in testing whether the area under the curve (AUC) derived from the risk prediction model is significantly greater than a cut-point of 0.6. Assuming a true AUC of 0.8 and flare rate of 50%, we will be able to reject the null hypothesis (H0: AUC ≤0.6) with 90% power and a 2-sided type I error rate of 0.025 with a target recruitment size of 100 patients.

### Recruitment of Patients

Patients will be recruited from the ROADMAP tapering clinic at the Freeman Hospital in Newcastle Upon Tyne, UK. Patients are referred to this National Health Service (NHS) by their usual rheumatology clinician, having identified that they have a clinical diagnosis of RA that is in remission and the patient is willing to consider DMARD tapering.

**Figure 1.**
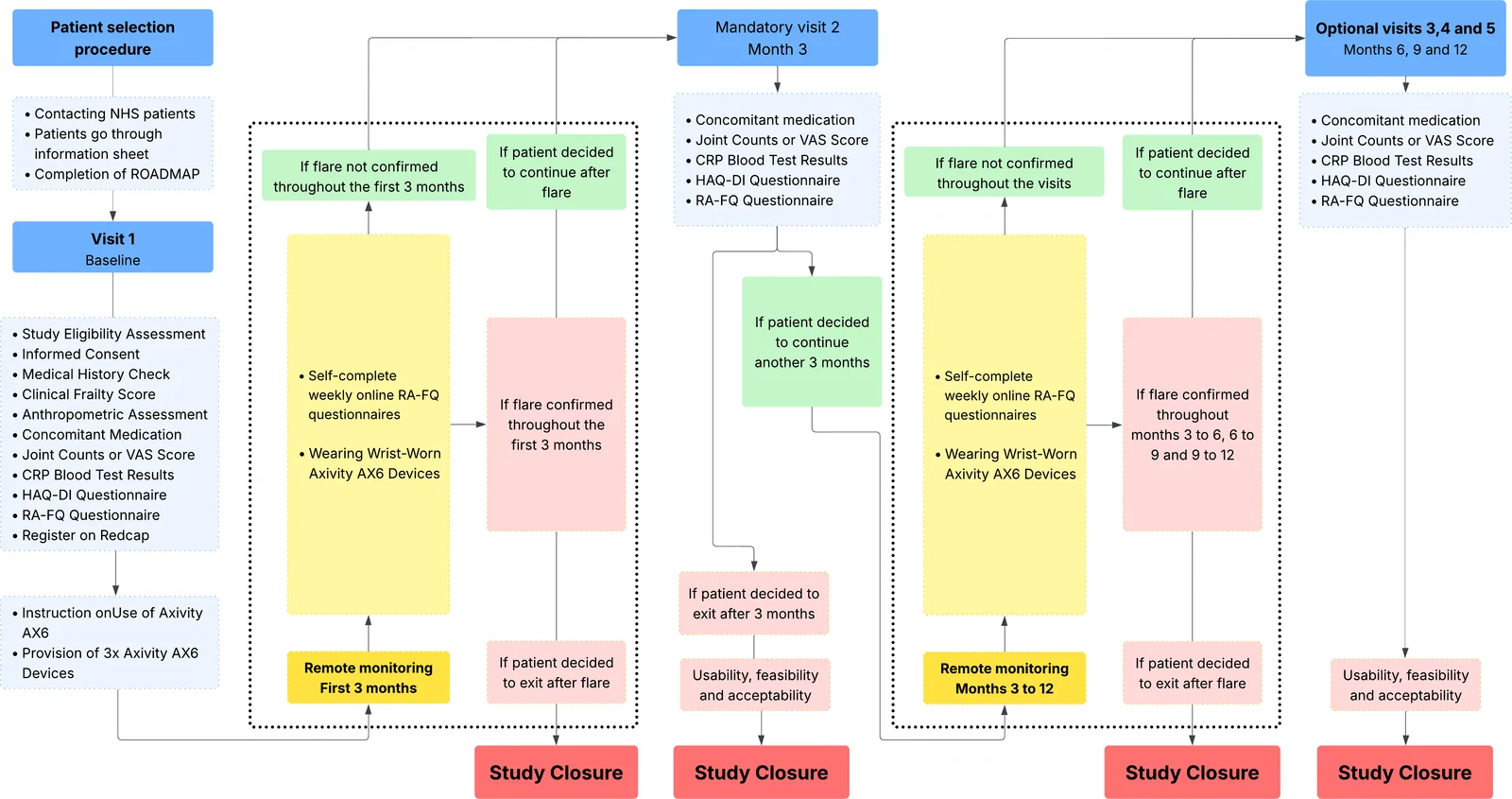
Overview of the study protocol, showing the planned study flow and key procedures across study phases. CRP: C-reactive protein; HAQ-DI: Health Assessment Questionnaire Disability Index; NHS: National Health Service; RA-FQ: Rheumatoid Arthritis Flare Questionnaire; ROADMAP: Rheumatoid Arthritis DMARD Tapering; VAS: visual analog scale.

Prior to their first ROADMAP clinic visit, patients will be posted the study participation information sheet to read beforehand (Figure 1). After completion of their first ROADMAP clinic visit, patients will be able to discuss the study further with a study investigator who can answer any questions they have about the study. Patients who would like to participate can then immediately enroll for their baseline study visit (ie, at the same hospital attendance as their ROADMAP appointment). Subsequent study visits are then scheduled to coincide with ROADMAP appointments to avoid additional clinic visits.

### Study Visits

#### Baseline Visit

Following the completion of the ROADMAP, patients attend baseline study visit (day 0). Patients attending their initial appointment will first undergo the inclusion and exclusion criteria check (Textbox 2).

Once the inclusion and exclusion criteria check is complete, patients will undergo the following study procedures in sequence. First, all patients will have the opportunity to discuss the study further with a study investigator before signing the consent form. Patients will next provide baseline information, including age, sex, address, telephone number, email address, past medical history, Clinical Frailty Score, Charlson Comorbidity Index, current medications, smoking status, and alcohol intake, after which height and weight will be recorded [16,17]. For each DMARD, the drug name and class, route, current dose and dosing frequency, reference therapeutic dose, proportion of the reference dose at enrollment, date and magnitude of the most recent dose reduction, number of previous tapering steps, and planned next tapering step will also be recorded. A researcher will then examine the patient to document the 28-joint tender and swollen counts, patient visual analog scale, and physician visual analog scale required for 28-joint Disease Activity Score with C-reactive protein (DAS28-CRP) disease activity assessment, and CRP blood test results obtained as part of routine clinical care during the ROADMAP clinic visit will also be recorded for DAS28-CRP calculation (Multimedia Appendix 2) [5,18].

Textbox 2. **Inclusion and exclusion criteria for study participation.**

**Inclusion criteria**

- Clinical diagnosis of rheumatoid arthritis (RA) made by a consultant rheumatologist
- 28-joint Disease Activity Score with C-reactive protein (DAS28-CRP)<2.4 at point of enrollment to the study
- Current (or expected within the next 28 days) tapering of a disease-modifying antirheumatic drug (DMARD; conventional synthetic, targeted synthetic, or biologic)
- Able to walk at least 4 m independently without walking aids
- Patient willing to commit to complete remote Rheumatoid Arthritis Flare Questionnaire (RA-FQ) and continuously wear monitoring devices

**Exclusion criteria**

- Current or recent (within past 12 months) use of rituximab
- Current or recent (within past 12 months) use of oral, intramuscular, intravenous, or intra-articular glucocorticoids for arthritis treatment (note that topical glucocorticoids and short courses of systemic glucocorticoids for other indications such as lung or skin conditions are permitted)
- Escalation of DMARD therapy (ie, initiation of new DMARD or increase in DMARD dose) within past 12 months (note that dose reductions are permitted)
- Unable to read or communicate in English
- Inability to provide informed consent
- Age less than 18 years
- Current diagnosis of a movement disorder
- Physical disability that would prevent wearing a wrist device (eg, bilateral upper limb amputation or congenital deformity)
- Current pregnancy

**Figure 2.**
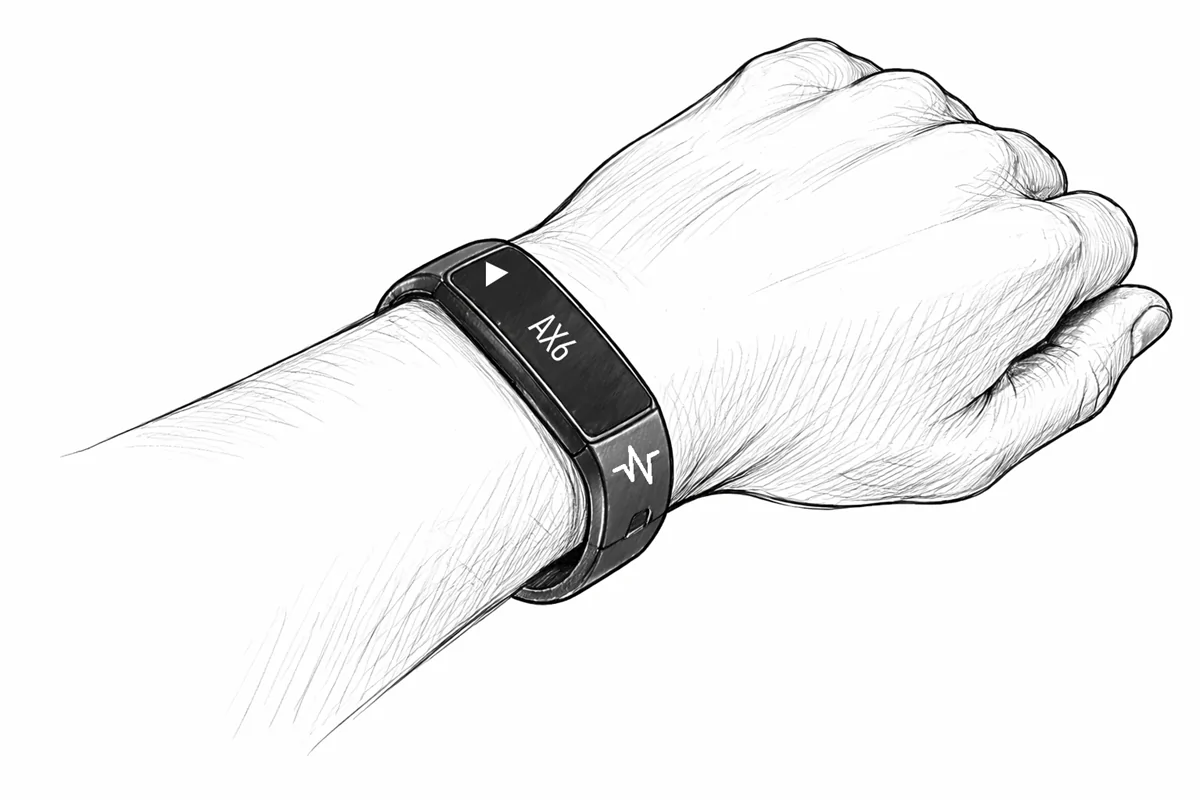
Participants will wear the device with the arrow on the device aligned with the arrow on the inside surface of the strap. The device was positioned so that the electrical pulse signal on the outside of the rubber strap faced the thumb, regardless of whether it was worn on the right or left wrist.

Patients will then complete the Health Assessment Questionnaire Disability Index (HAQ-DI) and the RA-FQ paper questionnaire [19]. They will subsequently be shown how to access the REDCap online portal to complete weekly online RA-FQ; if a patient cannot access the online platform because of limited internet or device access or limited digital literacy, they will be provided with a pack of 12 paper RA-FQs to complete weekly [11]. Patients will also be instructed, with written guidance, on how to wear the Axivity AX6 devices (Figure 2). Three Axivity AX6 devices and 1 wrist strap will be provided, and patients will wear each device on the nondominant wrist, or the dominant wrist if needed, in sequence for 28 days each, providing a continuous 3-month recording period. Finally, the study researcher will record whether the patient satisfies the ACR/EULAR 2010 RA Classification Criteria based on a retrospective review of clinical notes (Multimedia Appendix 3) [2].

#### Month 3 Visit (±1 Week)

At the week 12 visit, within a 1-week window, patients will be asked to verbally confirm their willingness to continue in the study, and any changes in concurrent medications and any adverse events (AEs) since the last clinic visit will be recorded. A researcher will then assess the 28-joint tender and swollen counts, patient visual analog scale, and physician visual analog scale required for DAS28-CRP disease activity assessment. The patient will complete an HAQ-DI questionnaire and an RA-FQ paper questionnaire, unless an online RA-FQ has already been completed within the previous 24 hours. Any paper questionnaires provided at the last visit and the Axivity AX6 devices from the previous study visit will be collected. If arthritis flare is confirmed, defined as a DAS28-CRP of at least 2.4 or at least 1 swollen joint, this will already have been treated appropriately during the concurrent ROADMAP clinic appointment, and the study investigator will record the patient’s estimated date of flare onset.

All patients will then be offered enrollment in the optional 12-week extension study. If the patient agrees, they will immediately enter the extension phase, be provided with 3 new Axivity AX6 devices and 1 wrist strap to wear for 28 days each in sequence on the nondominant wrist, or the dominant wrist if needed, and, if they are unable to access the online REDCap portal, they will also be given 12 additional paper RA-FQs to complete weekly; their next and final study visit will take place 12 weeks later, 84 days, according to schedule 6.3. If the patient declines the optional extension, they will exit the study at this visit and will be given a feedback questionnaire on the usability and acceptability of the AX6 devices together with a stamped addressed envelope for postal return to the study team.

#### Extension Study: Months 6, 9, and 12 Visits (±1 Week)—If Participating in Optional Extension Study

Participants entering the optional extension may attend follow-up visits at month 6 (week 24 ±1 wk), month 9 (week 36 ±1 wk), and month 12 (week 52 ±1 wk). At the month 6 and month 9 visits, participants will verbally confirm their willingness to continue. Changes in concurrent medications and any AEs since the previous visit will be recorded at each visit. A researcher will assess the 28-joint tender and swollen joint counts and the patient and physician visual analog scales. CRP results obtained through routine clinical care will also be recorded to calculate the DAS28-CRP. Participants will complete the HAQ-DI and a paper RA-FQ unless they have completed the online RA-FQ within the preceding 24 hours.

A clinically confirmed flare will be defined as a DAS28-CRP of ≥2.4 and/or at least 1 swollen joint attributable to inflammatory RA activity. Confirmed flares will be managed by the ROADMAP clinical team as part of routine care, and the study investigator will record the estimated date of flare onset. At each visit, completed paper questionnaires and Axivity AX6 devices from the preceding monitoring period will be collected. Participants who do not wish to continue at month 6 or month 9 will complete the wearable usability and acceptability questionnaire and exit the study. All participants remaining in the study at month 12 will complete the questionnaire and exit the study. No participant will be followed for more than 12 months from baseline. These optional extension visits will be implemented only after approval of the submitted ethics amendment and all required sponsor and site approvals.

#### Ad Hoc Flare Visits (Throughout the Study)

Patients tapering their DMARDs in the ROADMAP clinic may request additional ad hoc visits outside the usual 3-monthly schedules if they suspect an arthritis flare. There is no limit to the number of ad hoc visits, although in practice only one ad hoc visit would usually be expected for a patient who experiences a flare. In these cases, the patient will contact the study team to report possible flare symptoms, and a study investigator will then contact the ROADMAP clinic administrative team to arrange a ROADMAP clinic appointment and book a study visit to coincide with it. At the start of the ad hoc visit, patients will be asked to verbally confirm their willingness to continue in the study. Any changes in concurrent medications and any AEs since the last clinic visit will be recorded. A researcher will then assess the 28 joint tender and swollen counts, patient visual analog scale, and physician visual analog scale required for DAS28-CRP disease activity assessment. CRP blood test results obtained during routine clinical care at the ROADMAP clinic visit will also be recorded for DAS28-CRP calculation. The patient will then complete an HAQ-DI questionnaire and an RA-FQ paper questionnaire, unless an online RA-FQ has already been completed within the previous 24 hours.

If arthritis flare is confirmed, defined as a DAS28-CRP of at least 2.4 or at least 1 swollen joint, this will already have been treated appropriately during the concurrent ROADMAP clinic appointment. The study investigator will record the estimated date of flare onset, and follow-up in the main study period will be terminated if not already completed. Patients who are not already enrolled in the optional 12-week extension study will be offered enrollment. If they agree, they will immediately enter the extension phase, any paper questionnaires and Axivity AX6 devices from the last visit will be collected, and they will be provided with 3 new Axivity AX6 devices and 1 wrist strap to wear for 28 days each in sequence on the nondominant wrist, or the dominant wrist if needed. If they cannot access the online REDCap portal, they will also be given 12 additional paper RA-FQs to complete weekly, and their next study visit will take place in 12 weeks according to schedule 6.3, replacing the planned week 12 main study visit. If the patient is already enrolled in the optional extension study, they will be given a feedback questionnaire on AX6 usability and acceptability, will continue wearing the AX6 monitors and completing any provided paper RA-FQs for the remainder of the ongoing 12-week extension period, and will then exit the study without an extension week 12 review to avoid an extra visit outside the ROADMAP clinic schedule; they will also be given a stamped addressed envelope to return the AX6 monitors and paper questionnaires by post. If a patient with confirmed flare declines the optional extension study, they will exit the study at that visit, any paper questionnaires and AX6 devices from the last visit will be collected, and they will be given a feedback questionnaire on AX6 usability and acceptability together with a stamped addressed envelope for postal return to the study team. If arthritis flare is not confirmed, the patient will continue to wear the Axivity AX6 monitors and complete RA-FQs according to the current schedule and will attend the next study visit as already arranged.

### Patient Autonomy

Patients will have full autonomy throughout this study. The patient, informed of the full information as detailed in the patient information sheet, may withdraw their consent at any time. The wishes of the patient will be respected and would result in them exiting the study at that point. Any data collected up to that point would be retained and analyzed by the study team in line with the terms of the original patient consent form.

### Closure of the Study

Recruitment to the study will close upon recruitment of the 100th patient or on November 30, 2027, whichever is earlier. The end of the study is defined as the final visit of the last patient in the study.

### Delay to Final DAS28-CRP Calculation

As the DAS28-CRP calculation includes the CRP blood test result, the final DAS28-CRP score can only be fully calculated when the CRP result is available (ie, after conclusion of each study visit). Partial DAS28-CRP scores can be calculated at the time of the study visit and are usually indicative of final flare or remission status. However, it is possible that unexpected elevation of the CRP result may push the final DAS28-CRP score above the remission threshold and thus change the flare or remission status after the study visit has been completed. If this occurs at the baseline visit, then the patient would become ineligible to continue and should be withdrawn from the study at this point. If this occurs at a follow-up visit, the patient will continue with the Axivity AX6 and RA-FQ monitoring and follow-up schedule that was arranged in their recently completed study visit. Any necessary changes to clinical management will be arranged by the ROADMAP clinical team as per usual clinical care.

### Clinical Discretion

It is possible for DAS28-CRP scores to be elevated by events unrelated to inflammatory arthritis: for example, by a concurrent infection causing an elevation of CRP, or trauma causing a painful or swollen joint. The study investigator has discretion to discount such false elevations of the DAS28-CRP and follow the remission section for the relevant study visit if felt to be appropriate. In such cases, it is expected that the study investigator’s decision should match that of the ROADMAP service clinician; for example, if a patient with concurrent infection had an elevated DAS28-CRP, but the ROADMAP clinician considered their arthritis to still be in remission, then the study investigator should manage the patient as being in remission.

### Adverse Events and Serious Adverse Events

#### Adverse Event

An AE is defined as any untoward medical occurrence experienced by the patient during their participation within the study. The severity of AEs is defined as follows:

- Mild: Symptoms noted but no disruption to normal daily activities
- Moderate: Symptoms sufficient to disrupt normal daily activities
- Severe: Symptoms sufficient to prevent normal daily activities

#### Causality

All AEs should be assessed by the principal investigator or their medically qualified delegate for any causal relationship with a study procedure, namely, remote electronic patient-reported outcome measure (ePROM) or activity/physiological monitoring. Causality is defined in Table 1.

**Table 1.** Categories for assessing the causal relationship between an adverse event and trial medication.

Relationship	Description
Unrelated	There is no evidence of any causal relationship.
Unlikely	There is little evidence to suggest there is a causal relationship (eg, the event did not occur within a reasonable time after administration of the trial procedure). There is another reasonable explanation for the event (eg, the patient’s clinical condition, other concomitant treatment).
Possible	There is some evidence to suggest a causal relationship (eg, because the event occurs within a reasonable time after administration of the trial procedure). However, the influence of other factors may have contributed to the event (eg, the patient’s clinical condition, other concomitant treatments).
Probable	There is evidence to suggest a causal relationship and the influence of other factors is unlikely.
Definitely	There is clear evidence to suggest a causal relationship, and other possible contributing factors can be ruled out.
Not assessable	There is insufficient or incomplete evidence to make a clinical judgment of the causal relationship.

#### Serious Adverse Event

A serious adverse event (SAE) is any AE that results in death, is life-threatening (meaning the patient was at risk of death at the time of the event, not that the event might hypothetically have caused death if it had been more severe), requires hospitalization or prolongation of existing hospitalization, results in persistent or significant disability or incapacity, or is a congenital anomaly or birth defect. Clinical judgment should also be used to determine seriousness in other situations, and important AEs or reactions that are not immediately life-threatening and do not result in death or hospitalization but may jeopardize the patient or require intervention to prevent one of the outcomes listed above should also be classified as serious.

#### Expected Symptoms

This study will recruit patients with active RA, and as such are expected to have joint pain, joint swelling, joint stiffness, and fatigue, especially in the event of an arthritis flare.

#### Protocol Specifications

Joint pain, joint swelling, joint stiffness, and fatigue will not be recorded as an AE. An AE will not be classified as an SAE if it is a hospitalization, surgical procedure, or other medical intervention (whether as an inpatient, day-case, outpatient, or in primary care) that was planned before the recruitment of the patient to the study.

#### Reporting AEs and SAEs

AEs and SAEs will be reported as follows:

1. *All AEs* will be recorded on AE case report forms at the research site.
2. Site staff should report any *SAE immediately* to the chief investigator. Upon receipt of the SAE report, the chief investigator must:
  - *All SAEs*: Immediately inform the Sponsor (within 1 working day)
    - Inform the REC on behalf of the sponsor as soon as possible, and no later than 15 calendar days for nonfatal and non−life-threatening SAEs
  - *Unexpected SAEs related to a study procedure (suspected unexpected serious adverse reaction [SUSAR]):*
    - Inform the REC on behalf of the sponsor as soon as possible, and no later than 1 calendar day for fatal or life-threatening SAEs.
    - Convene a meeting of the chief investigator and study investigators as soon as possible, and no later than:
      1. Fifteen calendar days for fatal or life-threatening SAEs
      2. Thirty calendar days for nonfatal and non–life-threatening SAEs

#### Pregnancy

Pregnancy is associated with a diverse range of physiological changes and can therefore be expected to greatly influence physical activity data. Current pregnancy is an exclusion criterion because pregnancy-related physiological and behavioral changes may substantially affect physical activity and accelerometry-derived outcomes. If pregnancy is identified after enrollment, wearable monitoring and all further study-specific procedures will be discontinued, and the participant will be withdrawn from the study. The referring rheumatologist and general practitioner will be informed where clinically appropriate. Pregnancy will be documented and reported in accordance with sponsor safety-reporting requirements, and data collected before withdrawal will be retained and analyzed in accordance with the participant’s consent.

### Data Analysis

#### Preprocessing of Accelerometry Data

Axivity AX6 device data will be processed using established, standardized pipelines to derive quality metrics and daily summary metrics from the raw 3-dimensional acceleration and gyroscope signals, covering activity outcomes including volume (eg, step count), pattern (eg, distribution of activity across the day), and variability [20,21]; mobility outcomes including pace and rhythm variability [22]; and sleep outcomes including duration and sleep bouts, defined as interruptions [23].

#### Patient Acceptability

Quantitative feedback from patient acceptability questionnaire Likert scales will be summarized (median or mode and range). Anonymized free-text responses will be reproduced verbatim, analyzed for common themes, and presented in tabular form. The focus of these analyses is to gather information around patient acceptability for the purposes of improving the design of our future research studies, rather than to generate generalizable data and results suitable for a qualitative research publication [14,24].

### Statistical Analysis

#### Timing of Analyses

The main analysis will take place once follow-up has been completed for all participants and all data queries have been resolved, as far as possible.

#### Descriptive Analyses

##### Participant Baseline Characteristics, Clinical Measurements, and PROMs

Participant baseline characteristics, clinical measurements, and PROMs will be summarized based on the type of variable. Continuous variables will be described using appropriate summary statistics (eg, mean, SD and/or median, IQR, and range), and categorical variables will be presented as frequencies and percentages. Relevant plots will be generated to aid visualization.

##### RA-FQ

The RA-FQ is also designed to remotely monitor patients with RA during drug tapering. Participants rate, on a 0‐10 scale, their pain, difficulty with physical activities, fatigue, joint stiffness, and challenges in daily activities such as work, over the past week. At the end of the questionnaire, 2 yes or no questions ask whether the patient has experienced these symptoms for more than 1 week and whether they believe they are currently experiencing a flare of RA. The responses provide a standardized way to assess symptom severity and detect flares, helping clinicians track disease activity and adjust treatment if needed. The questionnaire will be collected weekly for each patient. For the 0‐10 scale items, the average score for each symptom will be calculated across all patients at 4 main visit time points, and summary statistics (eg, mean, SD, and/or median, IQR, and range) will be reported.

##### HAQ-DI Questionnaire

The HAQ-DI will be used to assess functional disability in patients with RA. It includes domains covering dressing and grooming, arising, eating, walking, hygiene, reach, grip, and activities or errands. Items are scored from 0 (no difficulty) to 3 (unable to do), with domain scores defined as the highest item score within each domain and adjusted for the use of aids or assistance according to standard scoring rules. The overall HAQ-DI score is calculated as the mean of domain scores (range 0‐3), with higher scores indicating greater disability. Scores will be summarized descriptively at each study visit using appropriate summary statistics (mean, SD, median, IQR, and range), and changes over time will be presented graphically.

##### Safety Outcomes

Safety outcomes, including AEs and SAEs, will be tabulated by type and severity, with the number and proportion of participants affected and the total number of occurrences reported.

### Primary Analyses

The primary outcome is the time from study enrollment to the first clinically confirmed RA flare, defined as a DAS28-CRP of 2.4 or above.

The primary objective of the study is to predict clinically confirmed flare risk using dynamic risk prediction methods based on daily accelerometry data including both the quality metrics and the physical activity measures. In addition, we would like to retrospectively assess the accuracy of the model vs patient-reported flare.

We will use landmarking models to develop a dynamic risk prediction approach for arthritis flare. Landmarking is a time-updated survival modeling framework that generates risk predictions at predefined time points (landmark times) using information available up to each time point. Predictions are then made over specified future time horizons.

The approach consists of two components:

1. A longitudinal submodel, which summarizes repeated accelerometry-derived measurements over time
2. A time-to-event submodel, which estimates the risk of flare from each landmark time

At each landmark time, the longitudinal submodel is used to extract summary measures of the accelerometry data up to that time, and these summaries are then included as predictors in the time-to-event model to estimate the future risk of flare.

The event of interest is the first clinically confirmed arthritis flare. Participants who do not experience a clinically confirmed flare will be censored at the end of their individual follow-up period or at earlier withdrawal.

For the time-to-event component, we will fit both Cox proportional hazards models and random survival forests (RSFs) to assess potential improvements in predictive performance and capture potential nonlinear relationships and interactions. We also consider including variables such as age, sex, Clinical Frailty Score, and anticitrullinated protein antibodies (ACPAs) antibody status (either positive/negative or the actual titer) as fixed effects or variables in both longitudinal and time-to-event submodels as they are clinically important.

The model will be generated at weekly landmark times throughout follow-up. At each landmark, the model will use all accelerometry data collected up to that time to estimate the probability of clinician-defined flare occurring within the subsequent 14 days. Weekly updating allows risk estimates to be refreshed throughout DMARD tapering and provides clinically actionable information to support decisions regarding continuation or modification of tapering.

We will also evaluate the accuracy of the model’s flare predictions for each prediction time window against patient-reported flares derived from the RA-FQ, based on responses to the yes or no question about current flare status. Patient-reported flare status will serve as the reference standard. Model-predicted flares will be compared against patient reports to calculate sensitivity, specificity, and agreement (Cohen κ). It helps to assess how well the model’s predictions align with patient perception.

- Sensitivity: proportion of patient-reported flares that are correctly predicted by the model (true-positive rate); higher values, close to 100%, are desirable.
- Specificity: proportion of patient-reported nonflares that are correctly identified as no flare by the model (true-negative rate); higher values, close to 100%, are desirable.
- Agreement: measured using Cohen κ to quantify concordance between model-predicted and patient-reported flares; higher values, close to 1, indicate stronger agreement beyond chance.

### Secondary Analyses

Our secondary objectives are to assess the usability and acceptability of continuous accelerometry monitoring using patient feedback questionnaires, retrospectively assess the accuracy of RA-FQ flare prediction vs clinically confirmed flare, and develop and evaluate a combined dynamic prediction model to investigate whether incorporating RA-FQ total scores alongside accelerometry-derived measurements improves the prediction of clinically confirmed flare.

Participant acceptability of continuous accelerometry monitoring will be assessed using a structured feedback questionnaire (Axivity Feedback Survey). The survey evaluates usability, comfort, technical issues, and impact on daily life (Section A: categorical and ordinal items), as well as overall satisfaction (0‐100 scale) and free-text comments (Section B).

Quantitative responses will be summarized descriptively. Categorical and ordinal items will be reported as frequencies and percentages, with graphical presentation where appropriate. The overall satisfaction score will be summarized using mean, SD, median, IQR, and range. Comparisons between time points (where applicable) may be explored using nonparametric tests. The free-text responses in Section B will not be analyzed, as they would require a qualitative analysis.

We also plan to refit the landmarking models for arthritis flare, using accelerometry data as longitudinal predictors and patient-reported flares from the RA-FQ as the outcome. This approach will help identify potential associations between accelerometry measures and patient-reported flares. Then, model-predicted flares will be compared against clinically confirmed flares to calculate sensitivity, specificity, and agreement (Cohen κ). This approach also allows us to assess how well the model’s predictions align with clinically confirmed flares.

Comparing the performance of these models with the ones mentioned in primary analyses will help assess whether accelerometry-derived signals are consistently predictive across different definitions of flare. If both models perform well, this will strengthen confidence that the signals are robust and not dependent on a single definition of flare.

As another secondary analysis, we will develop a combined dynamic prediction model to investigate whether incorporating patient-reported symptom information alongside accelerometry-derived measurements improves the prediction of clinically confirmed flare. The model will incorporate both longitudinal accelerometry-derived measures and the RA-FQ total score available at or before each prespecified landmark time. The RA-FQ captures patients’ subjective experiences of symptoms, including pain, stiffness, fatigue, and function, whereas accelerometry provides device-derived quality metrics and physical activity measures. The performance of the combined model will be compared with that of the accelerometry-only model using measures of discrimination and calibration to evaluate the additional predictive value of the RA-FQ.

### Missing Data

If any accelerometry-driven data are missing for a patient, the last observation carried forward (LOCF) method will be applied, as these data are collected daily and LOCF reflects the information available at the time of prediction. For the RA-FQ, if any item is missing for a patient, it will be replaced using the LOCF method, as the questionnaire is collected weekly.

For quantitative feedback from the participant acceptability questionnaire, missing responses in Section A will not be imputed, as they reflect participants’ perceptions at the time of data collection. For the overall satisfaction score (0‐100), missing values will also not be imputed, and analyses will be based on observed data only.

### Statistical Software

Statistical analyses will be carried out using the R software (version 4.4.3; R Core Team) and MATLAB (version 2025b; MathWorks, Inc.).

## Results

The first participant was recruited on October 9, 2025. As of July 1, 2026, a total of 16 patients have been recruited.

## Discussion

### Principal Findings

This protocol describes a prospective observational cohort study designed to evaluate whether remote monitoring can support earlier and more practical detection of RA flare during DMARD tapering in patients who are in remission at baseline. The study combines continuous wrist-worn accelerometry, weekly RA-FQ completion, and routine clinical assessments from the ROADMAP service to develop and evaluate a dynamic risk prediction model for clinically confirmed flare. The main expected contribution is to assess whether changes in digital activity and mobility patterns can predict the risk of flare before or around a clinically confirmed flare, using clinician-defined flare status and disease activity measures.

If successful, this study will provide early evidence that remote digital biomarkers can complement standard follow-up during tapering, when flare risk is clinically important and repeated in-person review may be difficult for patients and resource-intensive for services. The protocol is also designed to compare model-based prediction with patient-reported flare detection using the RA-FQ, which is important because both objective and subjective signals may provide distinct and clinically useful information.

### Comparison to Prior Works

This protocol is positioned within prior work on RA disease activity monitoring, remote monitoring implementation, PROM-based flare assessment, and wearable sensing. Traditional RA monitoring has relied on DAS-based assessment, including the DAS28 framework developed by Prevoo et al [5] and later remission-related agreement work by Fransen et al [7], but these approaches depend on in-person clinical review and laboratory testing. More recent rheumatology work has highlighted the value and practical challenges of remote monitoring in routine care, including service-level implementation experience reported by Watson et al [8] and implementation-focused recommendations discussed by Hamann et al [9]. In parallel, PROMs have become important for remote flare assessment, with the broader PROM evidence base summarized by Hendrikx et al [10], and the RA Flare Questionnaire specifically supported by the OMERACT work of Bartlett et al [11]. The wearable component of this protocol is also consistent with the growing literature on digital monitoring in rheumatic diseases, including the review by Davergne et al [12]. Importantly, this study directly extends our group’s prior RA accelerometry studies by Sarvestan et al [13,14], which demonstrated associations between wearable-derived metrics and inflammatory markers and showed feasibility and usability of wearable deployment in RA. The focus on flare prediction during DMARD tapering is clinically justified by evidence on flare risk during tapering or discontinuation summarized by Baker et al [6], Kuijper et al [15], and Rayner et al [25].

### Strength and Limitations

The main strength of this study is its embedding within an existing NHS DMARD tapering service, which improves real-world relevance and feasibility. Study visits are aligned with routine ROADMAP clinic appointments to reduce patient burden, and ad hoc flare visits are incorporated to capture clinically meaningful events when they occur. The protocol also includes practical accommodations for patients who cannot use the REDCap portal by offering paper RA-FQs, which may improve inclusivity and reduce avoidable exclusion related to digital access. Another strength is the multimodal design, combining clinician assessment, PROMs, and continuous wearable data. This supports richer phenotyping of flare-related change and may improve prediction performance compared with relying on any single data stream alone. The planned diagnostic evaluation using AUC, sensitivity, specificity, and predictive values is also appropriate for assessing clinical utility in an early development phase.

The protocol also has limitations. It is a single-site observational study, so external validity may be limited, and the findings may reflect local service pathways, patient selection, and tapering practices. The target sample is intended to support model development and pilot evidence rather than definitive effectiveness conclusions. In addition, flare classification depends partly on clinical review timing, which may introduce timing uncertainty in the reference outcome. The protocol acknowledges this and includes procedures for delayed final classification and investigator clinical discretion when the DAS28-CRP is elevated for reasons unrelated to inflammatory arthritis.

### Future Directions

The most important next step after this study will be external validation of the flare prediction model in larger and more diverse cohorts, ideally across multiple sites and health systems. Future work should also assess whether integrating wearable-based flare alerts into routine care improves patient outcomes, clinician workload, and service efficiency compared with standard tapering follow-up alone. In addition, model refinement may benefit from combining accelerometry and RA-FQ data directly in a unified prediction framework, as these data sources likely capture complementary aspects of flare onset and patient experience.

### Dissemination of Study Results

Study results will be presented at scientific conferences and/or published in peer-reviewed journals and patient group meetings. All personal identifiable information will be removed from the data during analysis to ensure that the results presented are anonymous. Data will be released from the study after a period of time necessary for the protection of any intellectual property rights.

## Supplementary material

10.2196/99594 Multimedia Appendix 1 Recommended disease-modifying antirheumatic drug tapering schedules as used in the Rheumatoid Arthritis DMARD Tapering clinic.

10.2196/99594 Multimedia Appendix 2 The 28-joint Disease Activity Score with C-reactive protein (DAS28-CRP) score.

10.2196/99594 Multimedia Appendix 3 2010 ACR/EULAR classification criteria for rheumatoid arthritis.

## Abbreviations

ACPA: anticitrullinated protein antibody
AE: adverse event
AUC: area under the curve
CRP: C-reactive protein
DAS28: 28-joint Disease Activity Score
DAS28-CRP: 28-joint Disease Activity Score with C-reactive protein
DMARD: disease-modifying antirheumatic drug
ePROM: electronic patient-reported outcome measure
ESR: erythrocyte sedimentation rate
HAQ-DI: Health Assessment Questionnaire Disability Index
LOCF: last observation carried forward
NHS: National Health Service
PROM: patient-reported outcome measure
RA: rheumatoid arthritis
RA-FQ: Rheumatoid Arthritis Flare Questionnaire
REC: Research Ethics Committee
ROADMAP: Rheumatoid Arthritis DMARD Tapering
RSF: random survival forest
SAE: serious adverse event
SUSAR: suspected unexpected serious adverse reaction
