# Supplementary material for: Remote Monitoring for Rheumatoid Arthritis Flare During Drug Tapering: Protocol for a Prospective Observational Cohort Study
Source: JMIR Res Protoc. 2026 Sep 21;15:e99594. doi: 10.2196/99594 (PMC13592738; doi:10.2196/99594)
Supplement: Supplementary file 1 [file resprot-v15-e99594-s001.docx]

**APPENDIX A: RECOMMENDED DMARD TAPERING SCHEDULES AS USED IN THE ROADMAP CLINIC.**

| DMARD | Step 1 | Step 2 | Step 3 |
| --- | --- | --- | --- |
| Methotrexate > 10mg once weekly | Greater of half dose1 or 10 mg once weekly | Stop | - |
| Methotrexate ≤ 10mg once weekly | Stop | - | - |
| Sulfasalazine > 1g daily | Half dose1 | Stop | - |
| Sulfasalazine ≤ 1g daily | Stop | - | - |
| Leflunomide | Stop | - | - |
| Hydroxychloroquine | Stop | - | - |
| JAK inhibitor | Half dose tablet2  (if available) | Every 2 days | Stop |
| Twice weekly biologic | Every week | Every 2 weeks | Stop |
| Once weekly biologic | Every 2 weeks | Every 3 weeks | Stop |
| Once fortnightly biologic | Every 3 weeks | Every 4 weeks | Stop |
| Once monthly biologic | Every 6 weeks | Every 8 weeks | Stop |
| 1 Rounding down to nearest multiple of available tablet dosage  2 Where a JAK inhibitor is usually prescribed twice daily (e.g. tofacitinib), then once daily dosing may be used as an alternative to halving the dose. | | | |
