# Supplementary material for: Remote Monitoring for Rheumatoid Arthritis Flare During Drug Tapering: Protocol for a Prospective Observational Cohort Study
Source: JMIR Res Protoc. 2026 Sep 21;15:e99594. doi: 10.2196/99594 (PMC13592738; doi:10.2196/99594)
Supplement: Supplementary file 2 [file resprot-v15-e99594-s002.docx]

**APPENDIX B: THE DISEASE ACTIVITY IN 28 JOINTS C-REACTIVE PROTEIN (DAS28-CRP) SCORE**

The DAS28-CRP score is calculated using the following parameters:

1. Swollen (SJC28) and tender (TJC28) 28 joint counts*

2. C-reactive protein (CRP, mg/L)

3. Patient global health assessment (VAS) (visual analogue scale, 0mm = best, 100mm = worst)

DAS28 (CRP)= 0.56√(TJC28) + 0.28√(SJC28) + 0.36ln(CRP[mg/l]+1) + 0.014(VAS[mm]) + 0.96

* = Where the 28 joints include the bilateral proximal interphalangeal joints, metacarpophalangeal joints, wrists, elbows, shoulders and knees.

Where CRP is below detectable levels, a value of zero will be used for the DAS28-CRP calculation.

The DAS28-CRP score has validated disease activity thresholds:

| Disease activity | DAS28-CRP threshold |
| --- | --- |
| Remission | < 2.4 |
| Low | 2.4 ≤ score ≤ 2.9 |
| Moderate | 2.9 < score ≤ 4.6 |
| High | > 4.6 |
