# Supplementary material for: Remote Monitoring for Rheumatoid Arthritis Flare During Drug Tapering: Protocol for a Prospective Observational Cohort Study
Source: JMIR Res Protoc. 2026 Sep 21;15:e99594. doi: 10.2196/99594 (PMC13592738; doi:10.2196/99594)
Supplement: Supplementary file 3 [file resprot-v15-e99594-s003.docx]

**APPENDIX C: 2010 ACR/EULAR CLASSIFICATION CRITERIA FOR RHEUMATOID ARTHRITIS**

Individual criteria may be cumulatively satisfied at any time point from onset of symptoms.

“Classification criteria for RA (score-based algorithm: add score of categories A–D; a score of 6/10 is needed for classification of a patient as having definite RA)”

|  | Score |
| --- | --- |
| A. Joint involvement |  |
| 1 large joint | 0 |
| 2-10 large joints | 1 |
| 1-3 small joints (with or without large joints) | 2 |
| 4-10 small joints (with or without large joints) | 3 |
| >10 joints (at least one small) | 5 |
|  |  |
| B. Serology |  |
| Negative RhF & negative CCP | 0 |
| RhF OR CCP >ULN but ≤ 3 x ULN | 2 |
| RhF OR CCP > 3 x ULN | 3 |
|  |  |
| C. Acute phase reactants |  |
| Normal CRP AND normal ESR | 0 |
| Abnormal CRP OR abnormal ESR | 1 |
|  |  |
| D. Duration of symptoms |  |
| <6 weeks | 0 |
| ≥6 weeks | 1 |

“’Large joints’ refers to shoulders, elbows, hips, knees, and ankles. ‘Small joints’ refers to the metacarpophalangeal joints, proximal interphalangeal joints, second through fifth metatarsophalangeal joints, thumb interphalangeal joints, and wrists.

ULN: upper limit of normal.
